# Diagnostic validation of a novel high-sensitivity cardiac troponin T assay

**DOI:** 10.1093/ehjacc/zuag044

**Published:** 2026-04-20

**Authors:** Ben Völschow, Bianca Strauß, Nils Arne Sörensen, Niklas Thießen, Jonas Lehmacher, Betül Toprak, Beya Abdennadher, Farnaz Dehkordi, Paul M Haller, Lea Scharlemann, Alina Schock, Astrid Petersmann, Friederike Gauß, Raphael Twerenbold, Johannes T Neumann

**Affiliations:** Department of Cardiology, University Heart and Vascular Center Hamburg, University Medical Center Hamburg-Eppendorf, Martinistrasse 52, Hamburg 20246, Germany; Department of Cardiology, University Heart and Vascular Center Hamburg, University Medical Center Hamburg-Eppendorf, Martinistrasse 52, Hamburg 20246, Germany; Department of Cardiology, University Heart and Vascular Center Hamburg, University Medical Center Hamburg-Eppendorf, Martinistrasse 52, Hamburg 20246, Germany; German Center for Cardiovascular Research (DZHK), Partner Site Hamburg/Kiel/Lübeck, Martinistrasse 52, Hamburg 20246, Germany; Department of Cardiology, University Heart and Vascular Center Hamburg, University Medical Center Hamburg-Eppendorf, Martinistrasse 52, Hamburg 20246, Germany; Department of Cardiology, University Heart and Vascular Center Hamburg, University Medical Center Hamburg-Eppendorf, Martinistrasse 52, Hamburg 20246, Germany; German Center for Cardiovascular Research (DZHK), Partner Site Hamburg/Kiel/Lübeck, Martinistrasse 52, Hamburg 20246, Germany; Department of Cardiology, University Heart and Vascular Center Hamburg, University Medical Center Hamburg-Eppendorf, Martinistrasse 52, Hamburg 20246, Germany; German Center for Cardiovascular Research (DZHK), Partner Site Hamburg/Kiel/Lübeck, Martinistrasse 52, Hamburg 20246, Germany; Department of Cardiology, University Heart and Vascular Center Hamburg, University Medical Center Hamburg-Eppendorf, Martinistrasse 52, Hamburg 20246, Germany; Department of Cardiology, University Heart and Vascular Center Hamburg, University Medical Center Hamburg-Eppendorf, Martinistrasse 52, Hamburg 20246, Germany; Department of Cardiology, University Heart and Vascular Center Hamburg, University Medical Center Hamburg-Eppendorf, Martinistrasse 52, Hamburg 20246, Germany; German Center for Cardiovascular Research (DZHK), Partner Site Hamburg/Kiel/Lübeck, Martinistrasse 52, Hamburg 20246, Germany; Department for Internal Medicine II, Division of Cardiology, Medical University of Vienna, Währinger Gürtel 18-20, 1090 Vienna, Austria; Department of Cardiology, University Heart and Vascular Center Hamburg, University Medical Center Hamburg-Eppendorf, Martinistrasse 52, Hamburg 20246, Germany; Department of Cardiology, University Heart and Vascular Center Hamburg, University Medical Center Hamburg-Eppendorf, Martinistrasse 52, Hamburg 20246, Germany; University Institute of Clinical Chemistry and Laboratory Medicine, University Oldenburg, Rahel-Straus-Straße 10, Oldenburg 26133, Germany; Institute of Clinical Chemistry and Laboratory Medicine, University Medicine Greifswald, Ferdinand-Sauerbruch-Straße, Greifswald 17489, Germany; University Institute of Clinical Chemistry and Laboratory Medicine, University Oldenburg, Rahel-Straus-Straße 10, Oldenburg 26133, Germany; Institute of Clinical Chemistry and Laboratory Medicine, University Medicine Greifswald, Ferdinand-Sauerbruch-Straße, Greifswald 17489, Germany; Department of Cardiology, University Heart and Vascular Center Hamburg, University Medical Center Hamburg-Eppendorf, Martinistrasse 52, Hamburg 20246, Germany; German Center for Cardiovascular Research (DZHK), Partner Site Hamburg/Kiel/Lübeck, Martinistrasse 52, Hamburg 20246, Germany; Department of Cardiology, University Heart and Vascular Center Hamburg, University Medical Center Hamburg-Eppendorf, Martinistrasse 52, Hamburg 20246, Germany; German Center for Cardiovascular Research (DZHK), Partner Site Hamburg/Kiel/Lübeck, Martinistrasse 52, Hamburg 20246, Germany; Department of Epidemiology and Preventive Medicine, School of Public Health and Preventive Medicine, Monash University, 553 St Kilda Road, Melbourne, VIC 3004, Australia

**Keywords:** Myocardial infarction, hs-cTnT, ACS, ESC 0/1h algorithm, Diagnostic accuracy

## Abstract

**Aims:**

High-sensitivity cardiac troponin (hs-cTn) assays are integral to evaluating suspected myocardial infarction (MI). A recently developed hs-cTnT assay requires diagnostic and prognostic evaluation.

**Methods and results:**

Patients presenting to the emergency department with suspected non-ST-elevation MI were prospectively evaluated. Discrimination was tested using the area under the curve (AUC). Diagnostic performance was assessed with previously derived cut-off concentrations and ESC 0/1 h algorithm. Head-to-head comparisons between the new and established assays were performed. Prognostic performance of hs-cTnT for 1-year mortality and major adverse cardiac events (MACE) was analysed using adjusted Cox regression. In 1415 patients, mean age 64 years and 64.2% male, MI was diagnosed in 12.1%. The novel TnT hs Gen 6 assay demonstrated excellent discrimination with an AUC on admission of 0.87 (95% CI: 0.84, 0.90) and 0.91 (95% CI: 0.89, 0.93) after 1 h. Using a 0 h cut-off <8 ng/L or <18 ng/L and Δ 0/1 h of <2 ng/L enabled rule-out in 54.3% (95% CI: 51.7%, 57.0%), with a NPV of 99.5% (95% CI: 98.7%, 99.9%); sensitivity was 97.7% (95% CI: 94.1%, 99.4%). For rule-in, a 0 h cut-off ≥112 ng/L or a Δ 0/1 h ≥ 10 ng/L yielded a PPV of 58.5% (95% CI: 51.9%, 64.9%) and specificity of 92.2% (95% CI: 90.6%, 93.6%) with rule-in of 16.5% (95% CI: 14.6%, 18.6%). Hs-cTnT was associated with 1-year mortality [hazard ratio (HR) 1.39 (95% CI: 1.28, 1.51)] and MACE [HR 1.22 (95% CI: 1.15, 1.29)].

**Conclusion:**

The novel hs-cTnT assay demonstrates high diagnostic accuracy to rule-out or diagnose MI and offers valuable prognostic information regarding 1-year mortality and MACE.

Trial Registration: clinicaltrials.gov (NCT02355457)

## Graphical Abstract

**Graphical Abstract:**
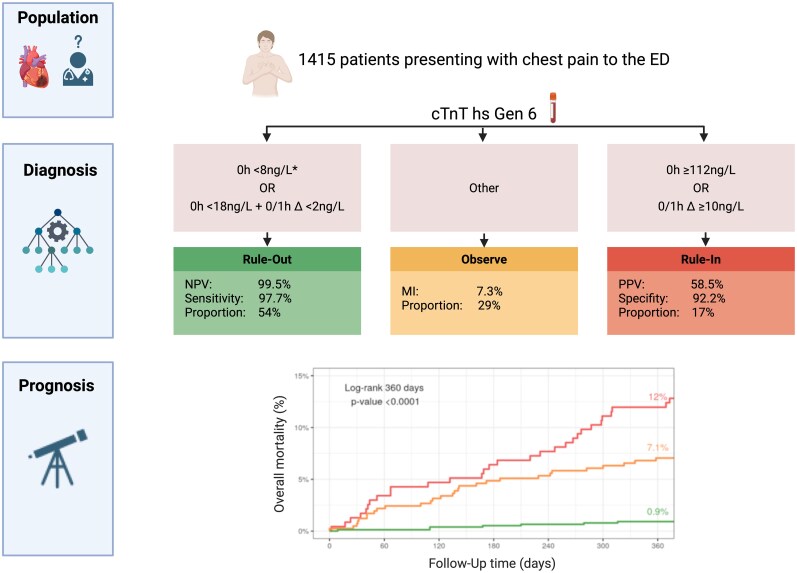
Diagnostic flow-chart of the 0/1 h ESC algorithm using the Roche TnT hs Gen 6 assay. Abbreviations: NPV, negative predictive value; PPV, positive predictive value; MI, myocardial infarction. * Chest pain onset >3 h.

## Introduction

Cardiovascular diseases, and especially myocardial infarction (MI), remain a leading cause of death and morbidity worldwide.^1,2^ Chest pain, the cardinal symptom for acute MI, is one of the most common symptoms for patients presenting to the emergency department (ED).^3,4^ High-sensitivity cardiac troponin (hs-cTn) assays are crucial for detecting myocardial injury in patients with suspected MI and are integral to the recommended 0/1 h or 0/2 h ESC diagnostic algorithms. Although several high-sensitivity troponin assays with assay-specific cut-offs have been validated and are recommended in the guidelines, further improvements in hs-cTn assays could optimize safe and rapid rule-out and rule-in of MI, enabling fast decision-making in routine clinical practice.^5,6^

Recently, the new TnT hs Gen 6 assay has been developed.^7^ However, data on the diagnostic and prognostic performance of this assay are very limited. A prior publication recommended newly derived assay-specific cut-off values for use in conjunction with the 0/1 h ESC algorithm. These cut-off concentrations achieved a rule-out rate of 56% with a sensitivity of 99.7% and rule-in rate of 20% with a specificity of 93.4%.^8^

Our aim was to validate the performance of this novel assay, including the recently derived cut-off values, in an independent study population and to evaluate its association with one-year outcomes, including mortality and major adverse cardiac events (MACE).

## Methods

### Study cohort and reference standard diagnosis

Patients from the prospective cohort study *Biomarkers in Acute Cardiac Care* (BACC) were used for these analyses. The BACC study has been published before.^6,9–11^ Briefly, patients with symptoms suggestive of MI presenting to the ED of the University Medical Center Hamburg-Eppendorf were enrolled. All patients were above 18 years old and provided written informed consent. For this analysis, patients with ST-elevation MI were excluded. Blood samples were collected directly at admission (0 h) and after 1 h (1 h). Furthermore, a 12-lead electrocardiogram was documented. When clinically indicated, further diagnostic and imaging was performed at the discretion of the treating physician. The final diagnosis for each patient was adjudicated independently by two physicians in a blinded fashion according to the 4th Universal Definition of MI, considering all available clinical data (i.e. laboratory data including serial measurements of the TnT hs Gen 5 assay by Roche Diagnostics and imaging data, as well as documented risk factors and pre-existing conditions assessed via the electronic patient record). In cases of incongruent adjudications, a third physician was consulted. Neither cardiologist was aware of the Roche Gen 6 hs-cTnT concentrations. For the established Elecsys® Troponin T hs Gen 5 assay by Roche Diagnostics, the test-specific limit of detection (LoD) was described as 3 ng/L. The 99th percentile has been reported as 13.5 ng/L in the general population in Europe (male 14.5 ng/L and female 10 ng/L) and as 19 ng/L in the general population in the United States (male 22 ng/L and female 14 ng/L). The coefficient of variation was 10% at 13 ng/L.^12,13^

The BACC study was registered at www.clinicaltrials.gov (NCT02355457) and approved by the local ethics committee; and complied with the Declaration of Helsinki.

### Study-specific measurements of the novel hs-cTnT assay

In addition to the collection of blood samples for routine clinical use, blood samples were also collected for biobanking directly at admission and after 1 h. Blood samples (lithium-heparin plasma tubes) were centrifuged, aliquoted, and stored frozen at −80°C under standardized conditions within 2 h. These samples were used for measuring hs-cTnT with the Elecsys® Troponin T hs Gen 6 assay by Roche Diagnostics at the University Institute of Clinical Chemistry and Laboratory Medicine in Oldenburg, Germany. For this assay, the 99th percentile was described as 27 ng/L (male 32 ng/L and female 18 ng/L).^14^ The corresponding coefficient of variation was 10% at 3.0 ng/L. The limit of detection was described as 1.5 ng/L.^7^ We found a CV% of 5.7% for the lowest quality control (3.21 ng/L).

For comparing the novel Troponin T hs Gen 6 assay with an alternative assay, the performance of the established Abbott Architect STAT hs-cTnI Assay was also tested regarding ROC curves. For the Abbott Architect STAT hs-cTnI Assay, a 99th percentile of 27 ng/L (male 33 ng/L and female 20 ng/L) and a limit of detection of 1.9 ng/L has been reported.^15^ The lowest cTnI concentration corresponding to a total CV of 10% was 5.6 ng/L.^16^

### Assessment of incident events

All patients were followed for at least 1 year to assess all-cause mortality as well as MACE after 1 year. Outcomes were collected by contacting patients by telephone, their general practitioner and/or evaluating the available medical records. If no direct contact was possible, the local register of death was contacted, and all cases of death were assessed.

### Statistical analysis

Continuous variables were described as median and quartiles (1st and 3rd), and categorical variables as absolute numbers and percentages. The Kruskal–Wallis test was used for continuous variables, and the *χ*^2^ test was employed for categorical variables for between-group comparisons. To evaluate the diagnostic performance for the novel hs-cTnT assay, troponin results from the 1415 above mentioned patients were used: Area under the curve (AUC), negative predictive value (NPV), positive predictive value (PPV), sensitivity, specificity, and the percentages of patients ruled out or ruled in for NSTEMI were calculated for every diagnostic algorithm. AUCs were compared using DeLong’s test.^17^ We evaluated prior derived cut-off concentrations for the TnT hs Gen 6 assay following the logic of the ESC 0/1 h algorithm. For rule-out of MI, a 0 h cut-off of <8 ng/L or a 0/1 h algorithm with a 0 h < 18 ng/L and a Δ 0/1 h change of <2 ng/L was used. For rule-in of MI, a 0 h cut-off ≥112 ng/L or a Δ 0/1 h change of ≥10 ng/L was used.^8^ Subgroup analyses were performed for individuals stratified by age, sex, symptom onset, and prevalence of coronary artery disease or renal disease.

Analyses for the predecessor TnT hs Gen 5 assay, following the logic of the ESC 0/1 h algorithm, were also performed to compare performances of the assays. For rule-out of MI, a 0 h cut-off of <5 ng/L or a 0/1 h algorithm with a 0 h < 12 ng/L and a Δ 0/1 h change of <3 ng/L was used. For rule-in of MI, a 0 h cut-off ≥52 ng/L or a Δ 0/1 h change of ≥5 ng/L was used.

For all-cause mortality and MACE Cox regression analyses with the TnT hs Gen 6 assay as the variable of interest was performed. The following sets of adjusting variables were used: (i) no adjustment, (ii) adjustment for age and sex, and (iii) adjustment for age, sex, any smoking, arterial hypertension, hypercholesterolaemia, diabetes mellitus, and family history of MI.

All statistical analyses were performed using R 4.2.2 (R Foundation for Statistical Computing).

## Results

### Patient characteristics

In total, 1415 patients were included, of whom 909 (64.2%) were male. The mean age was 64 years (IQR: 50–75 years) and mean BMI 26 kg/m^2^ (IQR: 24–30 kg/m^2^). Symptom onset <3 h when presenting to the ED was seen in 386 (29.3%) of all patients (*Table 1*). According to the 4th definition of MI, 102 (7.2%) patients had Type 1 NSTEMI, 69 (4.9%) type 2 NSTEMI, 44 (3.1%) an acute myocardial injury, and 375 (26.5%) a chronic myocardial injury. Patients with MI were significantly older (71 years vs. 62 years; *P* < 0.0001), had more often comorbidities like arterial hypertension (78.4% vs. 61.8%; *P* < 0.0001), hypercholesterolaemia (44.4% vs. 31.7%; *P* = 0.0012), and more often a history of MI (22.2% vs. 15.6%; *P* < 0.037) or coronary artery disease (CAD) (45.6% vs. 34.2%; *P* = 0.0044) when compared to non-MI patients.

**Table 1:** Patient characteristics for MI (NSTEMI Type 1 or Type 2) and non-MI

	Overall (*n* = 1415)	MI (*n* = 171)	Non-MI (*n* = 1244)	*P*-value
Age in years	64.0 (50.0, 75.0)	71.0 (59.0, 78.0)	62.0 (50.0, 75.0)	<0.0001
Male (%)	909 (64.2)	119 (69.6)	790 (63.5)	0.14
BMI in kg/m^2^	26.0 (24.0, 30.0)	26.0 (24.0, 30.0)	26.0 (24.0, 29.5)	0.61
Symptom onset < 3 h (%)	386 (29.3)	50 (31.2)	336 (29.0)	0.63
Heart Rate in b.p.m.	75.0 (65.8, 87.0)	79.5 (68.0, 94.0)	75.0 (65.0, 86.0)	0.0031
Actice smoker (%)	289 (20.7)	31 (18.8)	258 (21.0)	0.58
Former smoker (%)	317 (22.8)	48 (29.1)	269 (21.9)	0.049
**Comorbidities**				
Arterial hypertension (%)	901 (63.8)	134 (78.4)	767 (61.8)	<0.0001
Diabetes mellitus (%)	177 (12.7)	27 (15.8)	150 (12.2)	0.23
Hypercholesterolaemia (%)	470 (33.2)	76 (44.4)	394 (31.7)	0.0012
**History**				
History of CAD (%)	503 (35.5)	78 (45.6)	425 (34.2)	0.0044
History of MI (%)	232 (16.4)	38 (22.2)	194 (15.6)	0.037
Family history of MI (%)	270 (19.6)	38 (22.8)	232 (19.2)	0.33
**Laboratory**				
Estimated GFR in mL/min for 1.73 m^2^	77.0 (60.0, 91.7)	65.3 (48.8, 83.1)	78.7 (62.2, 92.6)	<0.0001
Hs-cTnT Gen 5 0 h in ng/L	9.0 (5.0, 20.0)	53.0 (20.0, 182.0)	8.0 (4.0, 16.0)	<0.0001
Hs-cTnT Gen 5 1 h in ng/L	9.0 (5.0, 20.1)	70.0 (30.2, 199.2)	8.0 (4.0, 15.0)	<0.0001
Hs-cTnT Gen 5 0h > limit of detection	1184 (85.1)	168 (98.2)	1016 (83.3)	<0.0001
Hs-cTnT Gen 5 0 h > overall 99th EU percentile	509 (36.6)	149 (87.1)	360 (29.5)	<0.0001
Hs-cTnT Gen 5 0 h > sex-specific 99th EU percentile	565 (40.6)	155 (90.6)	410 (33.6)	<0.0001
Hs-cTnT Gen 6 0 h in ng/L	13.3 (5.5, 40.2)	120.0 (38.6, 471.0)	11.0 (4.9, 29.2)	<0.0001
Hs-cTnT Gen 6 1 h in ng/L	13.5 (5.5, 41.3)	169.0 (59.6, 485.0)	11.0 (5.0, 28.0)	<0.0001
Hs-cTnT Gen 6 0 h > limit of detection	1385 (97.9)	171 (100)	1214 (97.6)	0.077
Hs-cTnT Gen 6 0 h > universal 99th percentile	478 (33.8)	144 (84.2)	334 (26.8)	<0.0001
Hs-cTnT Gen 6 0 h > sex-specific 99th percentile	544 (38.4)	149 (87.1)	395 (31.8)	<0.0001

Patient characteristics summarized by median (interquartile range in brackets) for continuous variables and by numbers (percentages in brackets) for binary variables. *P*-values were calculated using Kruskal–Wallis tests for continuous variables and *χ*^2^ tests for binary variables.

Abbreviations: BMI, body mass index; b.p.m., beats per minute; CAD, coronary artery disease; eGFR, estimated glomerular filtration rate; hs-cTnT, high-sensitivity cardiac troponin T; MI, myocardial infarction.

Coronary angiography was performed in 92.2% (94 of 102) of patients with Type 1 MI. Reasons for not performing coronary angiography varied and were due to individual reasons e.g. frailty, multimorbidity with reduced life expectancy, or conservative treatment at the patients’ request.

Using the TnT hs Gen 6 assay, 2.1% of patients had concentrations below the limit of detection (LOD), 64.1% of patients had concentrations between LOD and the 99th percentile, and 33.8% exceeded the 99th percentile (38.4% above the sex-specific 99th percentile). Using the TnT hs Gen 5 assay, 14.9% of patients had concentrations below the limit of detection (LOD), 48.5% of patients had concentrations between LOD and the 99th percentile, and 36.6% above the 99th percentile (40.6% above the sex-specific 99th percentile) (*Table 1* & Supplementary material online, *Table S1*).

### Diagnostic performance of the novel TnT hs Gen 6 assay

**Figure 1:**
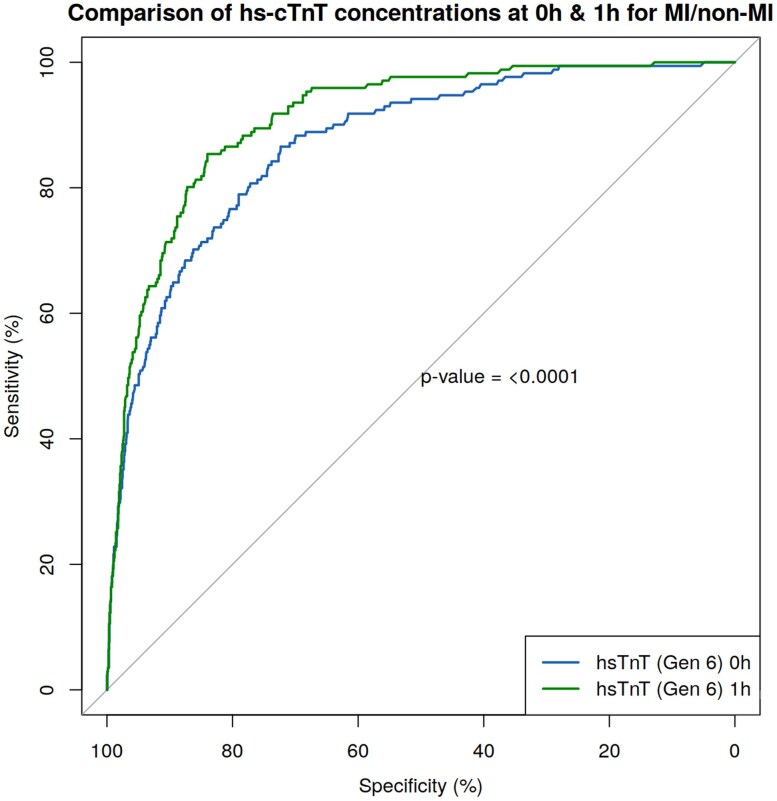
ROC curves for the diagnosis of MI compared to non-MI for 0 h and 1 h hs-cTnT. The Roche TnT hs Gen 6 assay showed high discriminatory ability for the diagnosis of MI with an AUC at 0 h of 0.87 (95% CI: 0.84, 0.90) and 0.91 (95% CI: 0.89, 0.93) after 1 h. Abbreviations: AUC, area under the curve; MI, myocardial infarction.

The TnT hs Gen 6 assay showed high discriminatory ability for the diagnosis of MI with an AUC at 0 h of 0.87 (95% CI: 0.84, 0.90) and an AUC at 1 h of 0.91 (95% CI: 0.89, 0.93) (*Figure 1*). Direct comparison with the TnT hs Gen 5 assay showed similar results with an AUC at 0 h of 0.87 (95% CI: 0.85, 0.90) and an AUC at 1 h of 0.91 (95% CI: 0.89, 0.94), as well as compared to the Architect STAT hs-cTnI Assay by Abbott with an AUC at 0 h of 0.88 (95% CI: 0.86, 0.91) and an AUC at 1 h of 0.92 (95% CI: 0.9, 0.94). When comparing the different assays, no statistical differences were seen by analyzing the differences in the ROC curves using DeLong’s test (see Supplementary material online, *Figure S1*).

### Validation rule-out and rule-in 0/1 h algorithm

Using the TnT hs Gen 6 assay and applying the previously derived cut-off concentrations for the 0/1 h rule-out algorithm yielded a NPV of 99.5% (95% CI: 98.7%, 99.9%) and sensitivity of 97.7% (95% CI: 94.1%, 99.4%), with 54.3% (95% CI: 51.7%, 57.0%) of patients classified as rule-out (*Table 2*). Importantly, all false-negative patients were classified as Type 2 MI (see Supplementary material online, *Table S2*).

**Table 2:** Diagnostic performance of the TnT hs Gen 6 assay using 0/1 h ESC algorithm for rule-out and rule-in of MI

	NPV (95% CI)	Sensitivity (95% CI)	Ruled-out % (95% CI)	1-year death (95% CI)	PPV (95% CI)	Specificity (95% CI)	Ruled-in % (95% CI)	1-year death (95% CI)	FN	TN	TP	FP	*n*
**Rule-out**													
0 h < 8 ng/L^a^	99.7 (98.2, 100)	99.4 (96.8, 100)	22.3 (20.2, 24.6)	1.0 (0, 2.0)					1	315	170	929	1415
0 h < 18 ng/L + 0/1 h Δ < 2 ng/L^b^	99.3 (98.1, 99.9)	96.4 (89.8, 99.2)	48.0 (44.8, 51.2)	0.9 (0.0, 1.7)					3	450	80	411	944
0 h < 8 ng/L^a^ OR (0 h < 18 ng/L + 0/1 h Δ < 2 ng/L)	99.5 (98.7, 99.9)	97.7 (94.1, 99.4)	54.3 (51.7, 57.0)	0.9 (0.2, 1.6)					4	765	167	479	1415
**Rule-in**													
0 h ≥112 ng/L					56.1 (47.9, 64.1)	94.5 (93.1, 95.7)	11.0 (9.4, 12.7)	13.5 (8.0, 18.8)	84	1176	87	68	1415
0/1 h Δ ≥10 ng/L^b^					63.3 (51.7, 73.9)	96.6 (95.2, 97.7)	8.4 (6.7, 10.3)	8.9 (2.4, 14.9)	33	832	50	29	944
0 h ≥112 ng/L OR 0/1 h Δ ≥10 ng/L					58.5 (51.9, 64.9)	92.2 (90.6, 93.6)	16.5 (14.6, 18.6)	12.0 (7.7, 16.0)	34	1147	137	97	1415

Abbreviations: CI, confidence interval; FN, false negative; FP, false positive; MI, myocardial infarction; NPV, negative predictive value; PPV, positive predictive value ;TN, true negative; TP, true positive.

^a^Chest pain onset >3 h.

^b^Subgroup: (No 0 h < 8 ng/L & chest pain onset >3 h) OR (NO 0 h ≥112 ng/L).

When applying the 0/1 h rule-out algorithm to the predecessor TnT hs Gen 5 assay, a NPV of 99.5% (95% CI: 98.7%, 99.9%) and sensitivity of 97.6% (95% CI: 94.1%, 99.4%) were observed, with 55.4% (95% CI: 52.8%, 58.1%) of patients classified as rule-out (see Supplementary material online, *Table S3*).

When applying the direct (0 h) rule-out algorithm, the Gen 5 assay showed a NPV of 99.5% (95% CI: 97.4%, 100.0%) and sensitivity of 99.4% (95% CI: 96.8, 100.0) with 15.6% (95% CI: 13.7%, 17.6%) of patients ruled-out. The Gen 6 assay demonstrated a similar NPV of 99.7% (95% CI: 98.2%, 100%) and sensitivity of 99.4% (95% CI: 96.8%, 100%) with a higher rule-out rate of 22.3% (95% CI: 20.2%, 24.6%) for direct (0 h) rule-out (*Table 2* & Supplementary material online, *Table S3*).

For the 0/1 h rule-in algorithm, the previously derived cut-off concentrations resulted in a PPV of 58.5% (95% CI: 51.9%, 64.9%) and specificity of 92.2% (95% CI: 90.6%, 93.6%), with 16.5% (95% CI: 14.6%, 18.6%) of patients classified as rule-in.

When applying the 0/1 h rule-in algorithm to the predecessor TnT hs Gen 5 assay a PPV of 58.2% (95% CI: 51.7%, 64.6%) and specificity of 91.8% (95% CI: 90.1%, 93.3%), with 17.2% (95% CI: 15.2%, 19.3%) of patients classified as rule-in was achieved (see Supplementary material online, *Table S3*).

**Figure 2:**
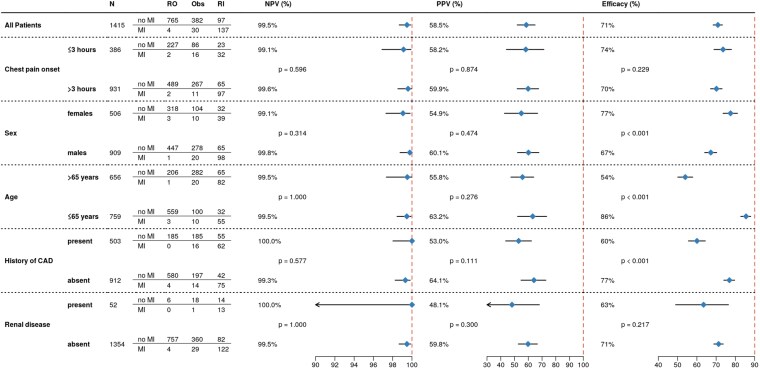
Subgroup analyses showing the diagnostic performance stratified by chest pain onset, sex, age, history of CAD, and renal disease. Abbreviations: CAD, coronary artery disease; MI, myocardial infarction; NPV, negative predictive value; Obs, observe; PPV, positive predictive value; RI, rule-in; RO, rule-out.

When applying both rule-out and rule-in criteria for the new TnT hs Gen 6 assay, 70.8% of patients were classified as either rule-out or rule-in, leaving 29.2% in the ‘observe’ group (*Graphical abstract*). In sensitivity analyses stratified by age, sex, symptom onset, and the prevalence of cardiovascular and renal disease, we observed consistent findings in all subgroups regarding PPV and NPV (*Figure 2*). When only focusing on patients with Type 1 NSTEMI, no MI was missed using the rule-out criteria, and the respective NPV was 100% (see Supplementary material online, *Table S4*).

### Prognostic evaluation

**Figure 3:**
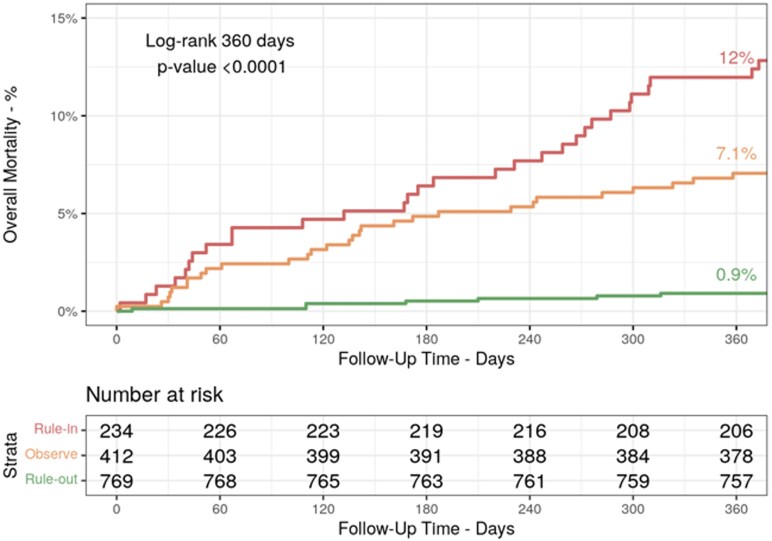
Kaplan–Meier curves for all-cause mortality stratified by group according to the 0/1 h algorithm.

Patients classified as rule-out had a low 1-year mortality rate of 0.9%, whereas patients classified as rule-in had a 1-year mortality rate of 12% (*Table 2* and *Figure 3*).

For the endpoint of all-cause mortality after 1 year, we performed Cox regressions with hs-cTnT measured by the TnT hs Gen 6 assay as the variable of interest on a logarithmic scale (*Table 3*). The hazard ratio (HR) for all-cause mortality for log hs-cTnT at 0 h was 1.55 (95% CI: 1.45, 1.65, *P* < 0.001) in an unadjusted model (Model 1). When adjusting for age and sex (Model 2), the HR was 1.40 (95% CI:1.30, 1.52, *P* < 0.001), and after adjusting additionally for smoking, arterial hypertension, hypercholesterolaemia, diabetes mellitus of any type and family history of CAD (Model 3) the HR was 1.39 (95% CI: 1.28, 1.51, *P* < 0.001).

**Table 3:** Cox regressions analyses for all-cause mortality and MACE for three different models with hs-cTnT measured by TnT hs Gen 6 assay as the variable of interest

	Model	HR (95% CI)	*P*-value	*n*	*n* events	EPV
**All-cause mortality**						
log of cTnT hs Roche Gen 6 0 h	1	1.55 (1.45–1.65)	<0.001	1415	220	220
	2	1.40 (1.30–1.52)	<0.001	1415	220	73.3
	3	1.39 (1.28–1.51)	<0.001	1356	212	26.5
**MACE**						
log of cTnT hs Roche Gen 6 0 h	1	1.35 (1.29, 1.41)	<0.001	1415	558	558
	2	1.24 (1.17, 1.31)	<0.001	1415	558	186
	3	1.22 (1.15, 1.29)	<0.001	1356	531	66.4

Model 1 = unadjusted; Model 2 = adjusted for age and sex; Model 3 = adjusted for Model 2 and smoking, arterial hypertension, hypercholesterolaemia, diabetes mellitus of any type, and family history of myocardial infarction/CAD.

Abbreviations: CAD, coronary artery disease; CI, confidence interval; EPV, events per variable; HR, hazard ratio; MACE, major adverse cardiac events.

Similarly, Cox regression analyses using the TnT hs Gen 5 assay showed that log hs-cTnT at 0 h was associated with all-cause mortality at 1 year with HRs of 1.65 (95% CI: 1.53, 1.79) in Model 1, 1.45 (95% CI: 1.32, 1.60) in Model 2, and 1.44 (95% CI: 1.30, 1.59) in Model 3 (see Supplementary material online, *Table S5*).

We also performed Cox regressions for the endpoint MACE after 1 year with hs-cTnT measured by the TnT hs Gen 6 assay as the variable of interest on a logarithmic scale. The HR for MACE was 1.35 (95% CI: 1.29–1.41, *P* < 0.001) in an unadjusted model. When adjusting for age and sex, the HR was 1.24 (95% CI: 1.17–1.31, *P* < 0.001), and after adjusting additionally for smoking, arterial hypertension, hypercholesterolaemia, diabetes mellitus of any type, and family history of CAD, the HR was 1.22 (95% CI: 1.15, 1.29, *P* < 0.001).

Cox regression analyses using the TnT hs Gen 5 assay showed that log hs-cTnT at 0 h was associated with MACE at 1 year with HRs of 1.42 (95% CI: 1.34, 1.50) in Model 1, 1.26 (95% CI:1.18, 1.35) in Model 2, and 1.24 (95% CI: 1.16, 1.33) in Model 3 (see Supplementary material online, *Table S5*).

## Discussion

In our analyses, we provide the first independent validation of cut-off concentrations for the new Roche TnT hs Gen 6 assay for ruling out or ruling in MI using a 0/1 h algorithm. We report two salient findings: First, the assay demonstrated a very high safety to rule-out MI, along with good discrimination between MI and non-MI cases. Second, we report a strong association of hs-cTnT concentrations with future cardiovascular events.

The study population, which was investigated in this study, is well established and has been a resource for many prior diagnostic studies. With 1415 patients enrolled and long-term follow-up available, the dataset is adequately powered allowing for risk factor stratification as well as analyses of outcome parameters.^18–21^ Our study cohort consists of an unselected population presenting to the ED of a large university hospital. Given the varying pre-test probabilities within this group, it can be considered broadly representative of the overall ED population in the western world. In our cohort, the distribution of sex and pre-existing comorbidities like arterial hypertension, hypercholesterolaemia, and diabetes mellitus was comparable to other similar studies, supporting the overall comparability of our findings.^22,23^ The MI prevalence in our cohort was 12.1%, which is in line with prior studies, where MI prevalence usually ranges between 5% and 15% among patients presenting to the ED with chest pain.^24–26^

Recently, the novel TnT hs Gen 6 assay was evaluated in the APACE cohort study. Here, the authors report excellent performance with high sensitivity and specificity, indicating safe and effective implementation of the ESC 0/1 h algorithm for rule-out of NSTEMI.^8^ In line with these findings, the discriminatory ability of the Roche TnT hs Gen 6 assay was also high in our present study and was comparable to other established troponin assays. When using the prior derived cut-off values for the 0/1 h algorithm from Koechlin *et al.* on behalf of the APACE study,^8^ the achieved NPV for rule-out of 99.5% and sensitivity of 97.7% are comparable to other assays allowing for safe use in clinical practice, as demonstrated by <1% death rate after 1 year. Of the overall cohort, only four patients were classified as false negatives, all of whom had Type 2 NSTEMI (two patients with supraventricular tachycardia, one patient with hypertensive urgency and one patient with atrial fibrillation, Supplementary material online, *Table S2*). When considering only Type 1 NSTEMI, the NPV and sensitivity for rule-out were both 100%. The proportion of 54.3% of patients being ruled-out is also satisfactory when considering rule-out rates between ∼48% and 65% in the literature for other hs-cTn assays using serial testing strategies.^18,19,27^

The achieved PPV of 58.5% and specificity of 92.2% using the prior derived cut-off values was relatively low compared to other studies. For the 0/1 h algorithm, PPVs between 63% and 75% for other hs-cTnT assays and PPVs between 62% and 83% for other hs-cTnI assays have been described with high dependence on the underlying MI prevalence and patient selection.^23,28,29^ This could be explained by differences in the enrolment strategies between the cohorts and differences in patient profiles. For example, the prevalence of MI in our cohort (12.1%) was lower than in the derivation cohort (18.4%). The proportion of ∼29% patients being in the ‘observe’ group was comparable to other assays and studies.^28–30^ However, in these patients, a higher rate of death (7.1% after 1 year) was observed, indicating an urgent need for individualized workup as recommended by recent guidelines.^5^ Importantly, the diagnostic performance remained robust in subgroup analyses by sex, age, symptom onset, prevalent CAD, or renal dysfunction.

Our findings confirm an excellent diagnostic capacity of the Roche TnT hs Gen 6 assay but also indicate that despite technological advancements in assay sensitivity and analytical performance, the incremental diagnostic value of new assays compared to already available assays may be limited.

With the new TnT hs Gen 6 assay, a higher proportion of patients had detectable hs-cTnT concentrations above the limit of detection, allowing detection of low-level hs-cTnT concentrations without apparent overdiagnosis of myocardial injury (see Supplementary material online, *Table S1*). However, while all patients with MI had hs-cTnT concentrations above the limit of detection at admission with the TnT hs Gen 6 assay, this was already the case for 98.2% of MI patients when using the TnT hs Gen 5 assay (*Table 1*). This aspect is underlined by similar results between TnT hs Gen 5 and Gen 6 assays regarding NPV, sensitivity, PPV, specificity, and rule-out/in rates.

Interestingly, in our subgroup analyses for the TnT hs Gen 6 assay, no interaction was seen between symptom onset <3 and ≥3 h in our cohort (*Figure 2*), indicating a potential use of the new TnT hs Gen 6 in early presenters. Importantly, no safety signal was observed in early presenters. However, the number of patients in this subgroup was limited, and further prospective studies evaluating different timepoints from symptom onset are needed to further address this finding.

Moreover, increased assay sensitivity is essential for cardiovascular risk prediction. Here, we demonstrate a strong association between Gen 6 hs-cTnT concentrations and prognostic outcomes like all-cause mortality and MACE after 1 year. This allows for risk stratification and allows individualized and prioritized care in the ED. This includes not only patients with a diagnosis of MI but also non-MI patients, as Cox regression analyses were performed without selecting for MI/non-MI.

A major strength of our analyses is the large and well-characterized dataset, which allows for rigorous diagnostic evaluation of the new assay. However, some limitations must be considered: Although our study is representative for the overall ED population in Germany, this was a monocentric study, and generalizability of our results is limited regarding ethnicity and other geographic regions. In the past, it has been reported that some assays were susceptible to interference from e.g. haemolysis and biotin, which might lead to falsely low troponin values, and these interferences can even have additive effects.^31^ Also, cardiac troponin-specific autoantibodies leading to macrotroponin have been described that can lead to prolonged elimination and reduced clearance, resulting in false-positive results.^32–34^ It must be noted that no interfering factors were tested in these analyses.

In conclusion, we report an excellent diagnostic accuracy of the Roche TnT hs Gen 6 assay in patients with suspected MI using a 0/1 h algorithm. Beyond diagnosis, it also provides valuable prognostic information regarding future cardiovascular events and all-cause mortality.

## Supplementary Material

zuag044_Supplementary_Data

## Data sharing

All data are incorporated into the article and its online supplementary material.
