# Supplementary material for: Diagnostic validation of a novel high-sensitivity cardiac troponin T assay
Source: Eur Heart J Acute Cardiovasc Care. 2026 Apr 20;15(7):535–44. doi: 10.1093/ehjacc/zuag044 (PMC13344171; doi:10.1093/ehjacc/zuag044)
Supplement: zuag044_Supplementary_Data [file zuag044_supplementary_data.docx]

**Supplementary Material**

**Figure S1:** Area under the Curve to discriminate MI and non-MI using different hs-cTn assays. AUC hs-cTnT Gen6 at 0h of 0.87 (95% CI 0.84,0.90) and at 1h of 0.91 (95%CI 0.89,0.93); AUC hs-cTnT Gen5 at 0h of 0.87 (95%CI 0.85, 0.90) and at 1h of 0.91 (95%CI 0.89, 0.94); AUC hs-cTnI STAT hs-cTnI Assay by Abbott at 0h of 0.88 (95% CI 0.86, 0.91) and at 1h of 0.92 (95% CI 0.9, 0.94).


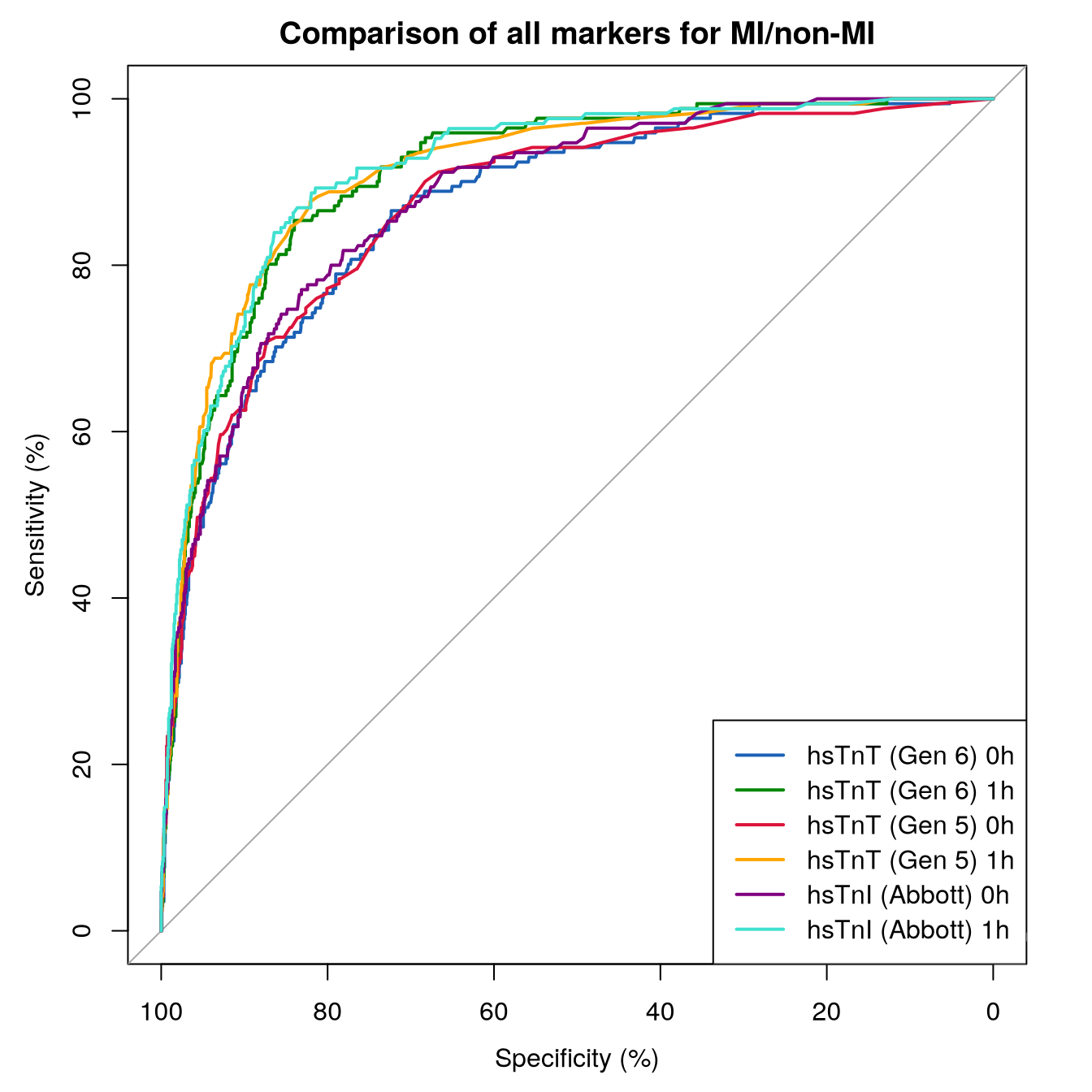


**Table S1:** Proportion of patients below LOD, between LOD and 99^th^ percentile and over 99^th^ percentile for MI (NSTEMI Type 1 or Type 2) and non-MI.

| **Laboratory** | Overall (N=1415) | MI (N=171) | Non-MI (N=1244) | p-value |
| --- | --- | --- | --- | --- |
| Hs-cTnT Gen 5 | | | | |
| Hs-cTnT Gen 5 0h in ng/L | 9.0 (5.0, 20.0) | 53.0 (20.0, 182.0) | 8.0 (4.0, 16.0) | <0.0001 |
| Hs-cTnT Gen 5 1h in ng/L | 9.0 (5.0, 20.1) | 70.0 (30.2, 199.2) | 8.0 (4.0, 15.0) | <0.0001 |
| Hs-cTnT Gen 5 0h ≤ limit of detection | 207 (14.9) | 3 (1.8) | 204 (16.7) | <0.0001 |
| Hs-cTnT Gen 5 0h between LOD and overall EU 99th percentile  (LOD ≤ Hs-cTnT Gen 5 0h < overall EU 99th percentile) | 675 (48.5) | 19 (11.1) | 656 (53.8) | <0.0001 |
| Hs-cTnT Gen 5 0h > overall EU 99th percentile (13.5 ng/L) | 509 (36.6) | 149 (87.1) | 360 (29.5) | <0.0001 |
| Hs-cTnT Gen 5 0h > sex-  specific EU 99th percentile | 565 (40.6) | 155 (90.6) | 410 (33.6) | <0.0001 |
| Hs-cTnT Gen 5 0h > overall US 99th percentile (14 ng/L) | 474 (34.1) | 145 (84.8) | 329 (27.0) | <0.0001 |
| Hs-cTnT Gen 5 0h > sex-  specific US 99th percentile | 586 (42.1) | 156 (91.2) | 430 (35.2) | <0.0001 |
| Hs-cTnT Gen 6 | | | | |
| Hs-cTnT Gen 6 0h in ng/L | 13.3 (5.5, 40.2) | 120.0 (38.6, 471.0) | 11.0 (4.9, 29.2) | <0.0001 |
| Hs-cTnT Gen 6 1h in ng/L | 13.5 (5.5, 41.3) | 169.0 (59.6, 485.0) | 11.0 (5.0, 28.0) | <0.0001 |
| Hs-cTnT Gen 6 0h ≤ limit  of detection | 30 (2.1) | 0 (0) | 30 (2.4) | 0.077 |
| Hs-cTnT Gen 6 0h between LOD and overall 99th percentile  (LOD ≤ Hs-cTnT Gen 6 0h < overall 99th percentile) | 907 (64.1) | 27 (15.8) | 880 (70.7) | <0.0001 |
| Hs-cTnT Gen 6 0h > overall 99th percentile | 478 (33.8) | 144 (84.2) | 334 (26.8) | <0.0001 |
| Hs-cTnT Gen 6 0h > sex-specific 99th percentile | 544 (38.4) | 149 (87.1) | 395 (31.8) | <0.0001 |

Patient characteristics summarized by median (interquartile range in brackets) for continuous variables and by numbers (percentages in brackets) for binary variables. P-values were calculated using Kruskal-Wallis tests for continuous variables and Chi-square tests for binary variables. Abbreviations: BMI = body mass index, MI = myocardial infarction, CAD = coronary artery disease, eGFR = estimated glomerular filtration rate, hs-cTnT = high-sensitivity cardiac troponin T, bpm = beats per minute.

**Table S2:** Characteristics of the four patients classified as false negative using the 0/1h ESC algorithm for rule-out of MI.

| **Clinical presentation & diagnostics** | | | | | | | | **Laboratory** | | | | | | | | **Diagnosis & clinical course** |
| --- | --- | --- | --- | --- | --- | --- | --- | --- | --- | --- | --- | --- | --- | --- | --- | --- |
| **Patient** | **Age** | **Sex** | **Symptoms** | **Symptom onset <3h** | **ECG & abnormal vital parameters** | **Pre-existing conditions** | **TTE** | **Gen 6 hs-cTnT 0h** | **Gen 6 hs-cTnT 1h** | **Gen 5 hs-cTnT 0h** | **Gen 5 hs-cTnT 1h** | **Gen 5 hs-cTnT 3h** | **Architect STAT**  **hs-cTnI 0h** | **Architect STAT**  **hs-cTnI 1h** | **Architect STAT**  **hs-cTnI 3h** |  |
| #1 | 58 | M | Recurrent palpitations together with first time angina pectoris | Yes | SVT  (HF = 190/min) | Paroxysmal atrial fibrillation | - | 5.6 | 6.9 | 7 | 7 | 19 | 3.6 | 3.3 | 18.9 | Type 2 NSTEMI due to SVT. Conversion after vagal maneuvers with discharge from ED on the same day. |
| #2 | 45 | F | Stabbing chest pain for several hours with joining palpitations and vomiting | No | AVNRT (HF= 188/min) with 2mm ST depression in II, III, aVF | No comorbidities known. Recurrent, self-limiting on-off palpitations for 5-30 minutes (~2x per month) | Normal findings | 2 | 3.1 | 2.9 | 3 | 9 | 2.9 | 1.8 | 12.9 | Type 2 NSTEMI due to AVNRT. Conversion after 6mg Adenosin. Discharge from ED on the same day on the patient’s request. Ambulatory follow-up with stress-ECG the following day with normal findings and no arrhythmias. |
| #3 | 54 | F | Chest pain during phone call with eye flickering and nausea | Yes | Normal ECG;  Blood pressure: Hypertensive (196/98mmHg) | None | Normal findings | 7.2 | 7.4 | 9 | 9 | 2.9 | 8.2 | 27.6 | 6.2 | Type 2 NSTEMI due to hypertensive urgency. After starting BP-medications and lowering of the blood pressure, symptoms disappeared; discharge from ED on the same day; Ambulatory follow-up performed. |
| #4 | 88 | F | Recurrent left thoracic chest pain with similar episode one year ago | No | Tachycardic atrial fibrillation (HF=110/min) | Atrial fibrillation with prior electrical conversion | Systolic LV-function in the lower normal range. No regional wall motion abnormalities. LA & RA dilated. Moderate TR. | 10.1 | 8.6 | 16 | 6 | 5 | 2 | 2.2 | 2.4 | Type 2 NSTEMI due to tachycardic atrial fibrillation. Successful electrical cardioversion. Coronary angiography performed with 50% LAD stenosis without need for intervention. Discharge after 3 days with planned ambulatory follow-up. |

Abbreviations: M=male, F=female; AF = atrial fibrillation; LA = left atrium; RA = right atrium; LV = left ventricle; TI = tricuspid valve regurgitation; SVT = supraventricular tachycardia; AVNRT = AV-nodal reentrant tachycardia**,** NSTEMI = Non-ST-segment elevation myocardial infarction; LAD = left anterior descending; ED = emergency department; BP = blood pressure; TTE = transthoracic echocardiography

**Table S3:** Diagnostic performance of the TnT hs Gen 5 assay using 0/1h ESC algorithm for rule-out and rule-in of MI

|  | **NPV (95% CI)** | **Sensitivity (95% CI)** | **Ruled-out % (95% CI)** | **1-year death (95% CI)** | **PPV (95% CI)** | **Specificity (95% CI)** | **Ruled-in**  **% (95% CI)** | **1-year death (95% CI)** | **FN** | **TN** | **TP** | **FP** | **N** |
| --- | --- | --- | --- | --- | --- | --- | --- | --- | --- | --- | --- | --- | --- |
| **Rule-out** | | | | | | | | | | | | |  |
| 0h <5ng/L* | 99.5  (97.4, 100.0) | 99.4  (96.8, 100.0) | 15.6  (13.7, 17.6) | 0.9  (0, 2.2) |  |  |  |  | 1 | 214 | 169 | 994 | 1378 |
| 0h <12 ng/L +  0/1h Δ <3 ng/L** | 99.5 (98.4, 99.9) | 96.3 (89.7, 99.2) | 54.0 (50.9, 57.1) | 0.9 (0.1, 1.7) |  |  |  |  | 3 | 546 | 79 | 388 | 1016 |
| 0h <5 ng/L* OR (0h <12ng/L + 0/1h Δ <3ng/L) | 99.5  (98.7, 99.9) | 97.6 (94.1, 99.4) | 55.4 (52.8, 58.1) | 0.9 (0.2, 1.6) |  |  |  |  | 4 | 760 | 166 | 448 | 1378 |
| **Rule-in** | | | | | | | | | | | | |  |
| 0h ≥52 ng/L |  |  |  |  | 59.2 (50.8, 67.2) | 95.0 (93.7, 96.2) | 10.7 (9.1, 12.4) | 11.6 (6.2, 16.6) | 83 | 1148 | 87 | 60 | 1378 |
| 0/1h Δ ≥5 ng/L** |  |  |  |  | 56.7 (45.8, 67.1) | 95.8 (94.3, 97.0) | 8.9 (7.2, 10.8) | 5.6 (0.7, 10.2) | 31 | 895 | 51 | 39 | 1016 |
| 0h ≥52 ng/L OR 0/1h Δ ≥5 ng/L |  |  |  |  | 58.2 (51.7, 64.6) | 91.8 (90.1, 93.3) | 17.2 (15.2, 19.3) | 9.3 (5.5, 12.9) | 32 | 1109 | 138 | 99 | 1378 |

Abbreviations: NPV = negative predictive value, PPV = positive predictive value, MI = myocardial infarction, FN = false negative, TN = true negative, TP = true positive, FP = false positive. CI = confidence interval, * Chest pain onset >3h; ** Subgroup: (No 0h <5ng/L & chest pain onset >3h) OR (NO 0h ≥52 ng/L).

**Table S4:** Diagnostic performance of Roche TnT hs Gen 6 assay using 0/1h ESC algorithm for rule-out of NSTEMI type 1

|  | **NPV**  **(95% CI)** | **Sensitivity**  **(95% CI)** | **Ruled-out %**  **(95% CI)** | **1-year death**  **(95% CI)** | **FN** | **TN** | **TP** | **FP** | **N** |
| --- | --- | --- | --- | --- | --- | --- | --- | --- | --- |
| **Rule-Out** | | | | | | | | | |
| 0h <8ng/L* | 100.0 (98.8, 100.0) | 100.0 (96.4, 100.0) | 22.3 (20.2, 24.6) | 1.0 (0, 2.0) | 0 | 316 | 102 | 997 | 1415 |
| 0h <18 ng/L +  0/1h Δ <2 ng/L** | 100.0 (99.2, 100.0) | 100.0 (88.8, 100.0) | 48.0 (44.8, 51.2) | 0.9 (0.0, 1.7) | 0 | 453 | 31 | 460 | 944 |
| 0h <8 ng/L* OR (0h <18ng/L + 0/1h Δ <2ng/L) | 100.0 (99.5, 100.0) | 100.0 (96.4, 100.0) | 54.3 (51.7, 57.0) | 0.9 (0.2, 1.6) | 0 | 769 | 102 | 544 | 1415 |

Abbreviations: NPV = negative predictive value, MI = myocardial infarction, FN = false negative, TN = true negative, TP = true positive, FP = false positive, CI = confidence interval, * Chest pain onset >3h; ** Subgroup: (No 0h <8ng/L & chest pain onset >3h) OR (NO 0h ≥112 ng/

**Table S5:** Cox regressions analyses for all-cause mortality and MACE for three different models with hs-cTnT measured by TnT hs Gen 5 assay as the variable of interest

|  | **Model** | **HR (95% CI)** | **p-value** | **N** | **N events** | **EPV** |
| --- | --- | --- | --- | --- | --- | --- |
| **All-cause mortality** | | | | | | |
| log of cTnT hs Roche Gen 5 0h | 1 | 1.65 (1.53, 1.79) | <0.001 | 1378 | 213 | 213 |
|  | 2 | 1.45 (1.32, 1.60) | <0.001 | 1378 | 213 | 71 |
|  | 3 | 1.44 (1.30, 1.59) | <0.001 | 1320 | 205 | 25.6 |
| **MACE** | | | | | | |
| log of cTnT hs Roche Gen 5 0h | 1 | 1.42 (1.34, 1.50) | <0.001 | 1378 | 543 | 543 |
|  | 2 | 1.26 (1.18, 1.35) | <0.001 | 1378 | 543 | 181 |
|  | 3 | 1.24 (1.16, 1.33) | <0.001 | 1320 | 517 | 64.6 |

Model 1 = unadjusted; Model 2 = adjusted for age and sex; Model 3 = adjusted for model 2 and smoking, arterial hypertension, hypercholesterinemia, diabetes mellitus of any type and family history of myocardial infarction/CAD. Abbreviations: MACE = major adverse cardiac events, HR = hazard ratio, CI = confidence interval, EPV = events per variable.
